# The psychological mechanism and heterogeneity of art design students’ intention to use AI: an empirical analysis based on an integrated framework

**DOI:** 10.3389/fpsyg.2026.1835335

**Published:** 2026-05-19

**Authors:** Chao Jiang, Bo Hui, Wen Sun, Jiazheng Li, Xuemei Wang, Ruonan Wang

**Affiliations:** 1 School of Fine Arts, Lianyungang Normal University, Lianyungang, China; 2 College of Design, Guangxi Arts University, Nanning, China; 3 School of Art and Design, Jiangsu Ocean University, Lianyungang, China; 4 College of Elementary Education, Capital Normal University, Beijing, China

**Keywords:** AI anxiety, art and design education, artificial intelligence (AI), creative self-efficacy, latent profile analysis (LPA), technology acceptance model (TAM)

## Abstract

The rapid development of Artificial Intelligence (AI) is profoundly reshaping the creative industries and art design education. Understanding the factors influencing students’ acceptance of Artificial Intelligence generated content is crucial for ensuring the sustainable application of this technology. This study investigated 630 Chinese university students majoring in art and design, adopting a combined variable-centered and person-centered approach to systematically examine the psychological mechanisms and heterogeneity of their intentions to use AI. Based on a serial mediation model, we hypothesized that general attitude toward AI itself and attitude toward using AI serve as mediators between distal antecedents (perceived usefulness, perceived ease of use, AI anxiety, creative self-efficacy) and AI usage intention. At the variable-centered level, hierarchical regression analysis and serial mediation analysis were used to test the predictive and indirect effects of perceived usefulness, perceived ease of use, AI anxiety, creative self-efficacy, general attitudes toward AI itself, and attitudes toward using AI on AI usage intention. At the person-centered level, latent profile analysis was employed to identify heterogeneous user groups based on AI usage intention across five design stages: creative ideation, material collection, visual element design, copywriting, and final modification and optimization. The study also examined differences in demographic and psychological variables across these profiles. Results indicated that the serial indirect effects of perceived usefulness, perceived ease of use, AI anxiety, and creative self-efficacy on usage intention via general attitude and usage attitude were significant, while their direct effects were non-significant, confirming full or partial mediation. General attitudes toward AI itself and attitudes toward using AI significantly and positively predicted usage intention in the hierarchical regression. Latent profile analysis identified three heterogeneous groups: Moderate Use Group, Low Use Group, and High Use Group. These three groups showed significant differences in grade level and key psychological variables, confirming the external validity of the profiles. The findings reveal the complexity of the psychological mechanisms underlying art design students’ AI usage intention and the heterogeneous nature of their usage patterns, providing a theoretical basis and practical implications for integrating AI tools and implementing differentiated teaching strategies in art design education within the Chinese context.

## 1 Introduction

The rapid development of Artificial Intelligence (AI) is profoundly reshaping the production methods and educational models of the creative industries (Zhu et al., 2025). From Midjourney and Stable Diffusion to DALL-E and Adobe Firefly, AI tools have evolved from assistive technologies to creative partners capable of image generation, copywriting, and creative ideation (Lyu et al., 2022). By fostering collaboration between human intuition and machine intelligence, these tools enable professional content creators and a broader audience to transcend traditional boundaries of “technique” and “efficiency,” exerting a profound impact on art education in higher education institutions (Chiu, 2024). A recent systematic review by Rong et al. (2025) pointed out that generative AI tools are being used in higher education to support ideation, multimodal expression, and interdisciplinary projects, which not only reshapes the technical logic of art production but also introduces new media, methods, and cognitive frameworks for art and design education. Research by Zhu et al. (2025) further confirms the growing importance of AI in art and design education, fundamentally changing students’ learning methods and creative collaboration models.

With the proliferation of AI technology, understanding the factors influencing students’ acceptance has become key to ensuring its sustainable application. Existing studies, based on theoretical frameworks such as the Technology Acceptance Model (TAM), the Unified Theory of Acceptance and Use of Technology (UTAUT), and the AI Device Use Acceptance (AIDUA) model, have explored users’ behavioral intentions towards AI-assisted design (Kelly et al., 2023). For example, Jiao and Cao (2024), integrating TAM and the Theory of Planned Behavior (TPB), investigated designers’ adoption of AI-assisted design, validating the model’s practical significance through data analysis from 392 Chinese designers. Li (2025), analyzing AI-assisted design usage intention from the perspectives of perceived anxiety, perceived risk, and UTAUT, found that performance expectancy, effort expectancy, social influence, and facilitating conditions significantly positively influenced usage intention, while perceived anxiety and perceived risk had negative effects. Regarding art and design students specifically, Hwang and Wu (2025) explored the impact of generative AI on design students’ creative cognition, discovering that self-efficacy and anxiety relief played a chain mediating role between AI and creative cognition. Research by Huang et al. (2024) also indicated that integrating AI into product design ideation teaching can significantly affect students’ self-efficacy and learning outcomes.

Although existing research has significantly advanced our understanding of AI tool acceptance, critical gaps remain when applying these findings to the specific context of art and design students.

First, recent studies have produced conflicting results regarding core TAM pathways in creative domains. While Li et al. (2026) found perceived usefulness (PU) to be a significant predictor for design students, Fan and Jiang (2024) reported non-significant effects for professional designers, and Zhou et al. (2024) observed that PU failed to predict behavioral intention in an AI-augmented design model. These inconsistencies suggest that purely cognitive models (PU, PEOU) may be insufficient to capture the unique tension between ‘tool empowerment’ and ‘identity threat’ (Hwang and Wu, 2025) experienced by student creators. Therefore, a more comprehensive framework is necessary—one that integrates not only cognitive appraisals but also emotional (AI anxiety), dispositional (creative self-efficacy), and meta-cognitive (general attitude toward AI) factors to resolve these contradictions.

Second, most existing studies adopt a variable-centered approach (e.g., Jiao and Cao, 2024; Zhu et al., 2025), which assumes sample homogeneity and reports average effects. While valuable, this approach cannot identify qualitatively different user profiles—a critical oversight given that educational interventions must be tailored to heterogeneous student needs. Emerging person-centered research in adjacent fields (Chen and Zare, 2025) has revealed distinct latent profiles of AI thinking, yet no study has applied this approach to map art and design students’ stage-specific AI usage intentions. The present study fills this methodological and theoretical gap by combining variable-centered serial mediation (to test the ‘how’ of psychological influence) with person-centered latent profile analysis (to identify ‘who’ needs what kind of support), thereby offering a more complete and actionable understanding.

### 1.1 The psychological mechanism perspective: a variable-centered framework of AI usage intention

Understanding individuals’ acceptance of emerging technologies has long been a central issue in information systems research. With the rapid development of generative AI in creative domains, explaining users’ usage intention requires moving beyond a purely functionalist perspective. In particular, AI design tools are not only instruments for task completion but also active agents that participate in creative processes. As a result, users’ responses involve not only cognitive evaluations but also emotional reactions, self-related beliefs, and broader attitudes toward AI.

To capture this complexity, the present study adopts a variable-centered psychological framework that integrates four dimensions: cognitive evaluations (perceived usefulness and perceived ease of use), emotional responses (AI anxiety), trait-level characteristics (creative self-efficacy), and meta-cognitive attitudes toward AI. This framework provides a more comprehensive account of art and design students’ acceptance of AI design tools.

#### 1.1.1 Extension of a classic framework: TAM in the context of AI design tools

The Technology Acceptance Model (TAM; Davis, 1989) provides a foundational framework for understanding technology usage intention, with perceived usefulness (PU) and perceived ease of use (PEOU) as its core determinants. Extensive research has validated TAM across diverse technological contexts, consistently showing that PU is the strongest direct predictor of behavioral intention, while PEOU influences intention both directly and indirectly through PU (Granić and Marangunić, 2019; Davis et al., 1989).

Recent studies have extended TAM to the context of generative AI, confirming its general applicability in design and educational settings. Empirical evidence consistently shows that both PU and PEOU significantly predict users’ intention to adopt AI tools (e.g., Yao et al., 2025; Li et al., 2026; Zou and Huang, 2023; Romero-Rodríguez et al., 2023).

However, AI design tools differ from traditional technologies in that they function as non-human agents with generative and creative capabilities. This introduces experiences that go beyond instrumental utility, particularly in creative domains where users may experience a tension between “tool empowerment” and “identity threat” (Hwang and Wu, 2025; Alsuwaida and Alsamiri, 2025; Marrone et al., 2022).

Moreover, recent studies have identified boundary conditions of TAM in AI contexts. Some findings suggest that traditional predictors such as PEOU or PU may become less dominant, while factors such as enjoyment, perceived value, and emotional responses play increasingly important roles (Fan and Jiang, 2024; Zhou et al., 2024).

Taken together, these findings indicate that while TAM provides a useful foundation, it should be extended by incorporating emotional, trait-level, and meta-cognitive variables to better capture AI acceptance in creative contexts.

#### 1.1.2 The emotional dimension: AI anxiety

Traditional technology acceptance models have primarily emphasized rational cognitive evaluations, often overlooking emotional influences. However, as AI systems increasingly act as creative collaborators rather than passive tools, emotional responses—particularly anxiety—have become critical for understanding technology adoption.

AI anxiety refers to individuals’ concerns about technological substitution, capability devaluation, and rapid technological change (Johnson and Verdicchio, 2017). It has been conceptualized as a multidimensional construct, including learning-related anxiety, job replacement concerns, and broader ethical or societal worries (Wang and Wang, 2022; Wang and Xiao, 2025).

In creative domains, AI anxiety is especially salient because of its close association with identity-related concerns. AI tools can simultaneously enhance productivity and threaten individuals’ sense of authorship and professional value, creating a tension between “tool empowerment” and “identity threat” (Hwang and Wu, 2025; Dasari, 2025).

Importantly, AI anxiety is not purely inhibitory. Moderate levels of anxiety may promote reflection and adaptive learning. Empirical research shows that self-efficacy can alleviate anxiety and indirectly enhance creative performance, highlighting the interaction between emotional and cognitive mechanisms (Hwang and Wu, 2025; Li, 2025). Furthermore, training experiences may reduce task-related anxiety while simultaneously increasing concerns about long-term professional implications, indicating the dynamic nature of this construct (Ağca and Korkmaz, 2025).

Overall, AI anxiety in art design education can be characterized by three key features: multidimensionality, reflecting diverse sources of concern; dynamism, as different forms of anxiety evolve with experience; and contextuality, with identity-related concerns playing a central role. Incorporating AI anxiety into the present framework therefore extends TAM beyond purely cognitive evaluations and enriches our understanding of students’ responses to AI tools.

#### 1.1.3 The trait dimension: creative self-efficacy

In addition to situational cognitive and emotional factors, stable individual differences also play a critical role in technology acceptance. Creative self-efficacy (CSE), derived from Bandura’s (1977) self-efficacy theory, refers to individuals’ beliefs in their ability to produce creative outcomes (Tierney and Farmer, 2002).

In educational research, CSE has been consistently shown to predict creative performance, persistence, and engagement in creative tasks (Unal and Tasar, 2021; Yang and Hsu, 2020).

In the context of AI-assisted design, CSE plays a particularly important role. It not only directly promotes creative cognition but also interacts with emotional factors. For example, higher self-efficacy can reduce anxiety and increase individuals’ willingness to engage with AI tools, thereby facilitating creative outcomes (Hwang and Wu, 2025; Huang and Kou, 2026). At the same time, instructional interventions integrating AI have been shown to enhance both CSE and creative performance, although effects may differ across creative dimensions (Huang et al., 2024; Li and Jiang, 2025).

From a theoretical perspective, CSE can be developed through multiple pathways, including mastery experiences, observational learning, and social persuasion (Jobst and Meinel, 2012), and may buffer the negative effects of external evaluation in creative contexts (Hu et al., 2018).

Overall, CSE functions as a key psychological mechanism linking external learning environments with internal cognitive and emotional processes. Compared with situational variables such as PU and PEOU, it reflects deeper beliefs about one’s creative capability and provides stronger explanatory power for sustained engagement with AI tools.

#### 1.1.4 The meta-cognitive dimension: attitude towards artificial intelligence

Beyond cognitive evaluations, emotional responses, and trait-level characteristics, individuals’ general attitudes toward AI represent an important meta-cognitive dimension influencing technology acceptance. These attitudes reflect broader and more stable evaluations of AI’s role in creativity and society, shaping how individuals interpret and interact with AI tools across contexts.

One central dimension concerns the perceived artistic value of AI-generated content. Research shows that individuals tend to evaluate artworks less favorably when they believe they are created by AI rather than humans, reflecting underlying beliefs about the legitimacy of AI as a creative agent (Hong and Curran, 2019; Bao, 2025; Latikka et al., 2023).

Another dimension involves ethical and societal considerations, including concerns about intellectual property, fairness, and data use. These issues play an important role in shaping trust in AI and influencing adoption decisions (Wang, 2024; Schauer et al., 2025).

A third dimension relates to individuals’ understanding of the relationship between AI and human creativity. Users often hold ambivalent perspectives, recognizing AI’s benefits for efficiency and inspiration while expressing concerns about dependency, reduced originality, and identity loss (Sayilgan et al., 2025; Kaila et al., 2025).

Importantly, these attitudes have been shown to influence both learning behaviors and creative outcomes. Positive attitudes are associated with greater engagement and acceptance, whereas negative attitudes may lead to resistance or critical evaluation of AI outputs (Kim et al., 2024; Sáez-Velasco et al., 2025; Xue, 2026). Moreover, such attitudes are shaped by experience, disciplinary background, and instructional context (Alsuwaida and Alsamiri, 2025; Akman, 2025).

Overall, attitudes toward AI function as a higher-order cognitive framework that integrates value judgments, ethical considerations, and beliefs about creativity. Incorporating this meta-cognitive dimension complements cognitive, emotional, and trait-level variables, providing a more comprehensive understanding of AI acceptance in creative education.

### 1.2 The person-centered perspective: heterogeneity in AI usage intention and refined measurement

The variable-centered perspective reviewed above examines relationships between psychological variables and AI usage intention under the assumption of sample homogeneity, typically focusing on average effects derived from regression-based approaches (Chen and Zare, 2025). While this approach is effective for identifying key predictors, it is limited in capturing the heterogeneity of individuals—specifically, the possibility that qualitatively distinct subgroups exist with different configurations of psychological characteristics and behavioral intentions.

To address this limitation, the person-centered perspective has gained increasing attention. This approach treats the individual as the unit of analysis and aims to identify latent subgroups based on patterns across multiple variables. Latent Profile Analysis (LPA), a widely used person-centered method, has been successfully applied in technology-related research. For example, Chen and Zare (2025) identified four distinct profiles of AI-related thinking among university students, demonstrating that individuals differ not only in levels of engagement but also in the configuration of cognitive and emotional characteristics.

Evidence from related domains further supports the relevance of this perspective. In art experience research, heterogeneous experiential profiles have been identified across individuals, with distinct patterns linked to differences in evaluation, decision-making, and wellbeing outcomes (Miller et al., 2025). These findings suggest that, in art and design education, students’ AI usage intentions may similarly exhibit meaningful heterogeneity rather than forming a single continuum.

Building on this perspective, the present study assumes that art and design students may demonstrate qualitatively different patterns of AI usage intention. Identifying such subgroups is essential for understanding the formation of usage intention and for informing differentiated instructional strategies.

A key prerequisite of this research pathway is the use of refined measurement. Existing studies, such as Zhu et al. (2025), have measured students’ behavioral intention toward AI usage using global, non-context-specific scales, without differentiating between specific stages of the design process. Such global measures may overlook important contextual differences. However, AI involvement can vary substantially across different stages of the design process—such as ideation, material collection, visual design, copywriting, and final optimization. Global measures therefore fail to capture this variability and may obscure meaningful individual differences.

To address this limitation, the present study adopts a task-based, contextualized measurement approach. The decomposition of the design process into core stages draws on established models in design research. For example, Ambrose and Harris (2015) proposed a seven-stage framework for visual communication design (Define–Research–Ideate–Prototype–Select–Implement–Learn), conceptualizing design as a structured process from problem definition to implementation and reflection. Considering the characteristics of generative AI-assisted creative tasks in art and design education, the present study operationalizes the design process into five core stages: creative ideation, material collection, visual element design, copywriting, and final modification and optimization. For each stage, students are asked to indicate the proportion of work they would prefer AI to complete. This approach shifts the focus from general attitudes to concrete task allocation decisions, thereby improving ecological validity and providing more fine-grained data for identifying heterogeneous usage patterns.

Integrating contextualized measurement with LPA enables a systematic research pathway from refined measurement to subgroup identification and subsequent validation. Accordingly, the second research objective of this study is to identify latent profiles of students based on their AI usage intentions across different design stages, characterize the distinct patterns of AI use, and examine differences across profiles in demographic and psychological variables.

### 1.3 The current study

Building on the critical gaps identified above, the present study advances existing knowledge in three distinct ways: (a) theoretically, by proposing and testing a serial mediation model that integrates cognitive, emotional, and meta-cognitive antecedents within a unified framework; (b) methodologically, by introducing a task-allocation measure of AI usage intention across five design stages, which provides higher ecological validity than global scales; and (c) analytically, by combining variable-centered (serial mediation) and person-centered (latent profile analysis) approaches to uncover both universal mechanisms and heterogeneous user patterns.

This integrated model posits that all four distal antecedents (PU, PEOU, AI anxiety, and CSE) do not directly influence AI usage intention. Instead, their effects are fully channeled through a sequential attitudinal chain: first, they shape students’ general attitude toward AI itself (M1); second, this general attitude influences their attitude toward using AI (M2); and third, attitude toward using AI drives AI usage intention (Y). Thus, the model explicitly rejects the notion of independent direct effects and instead proposes a unified serial mediation framework.

**Figure 1:**
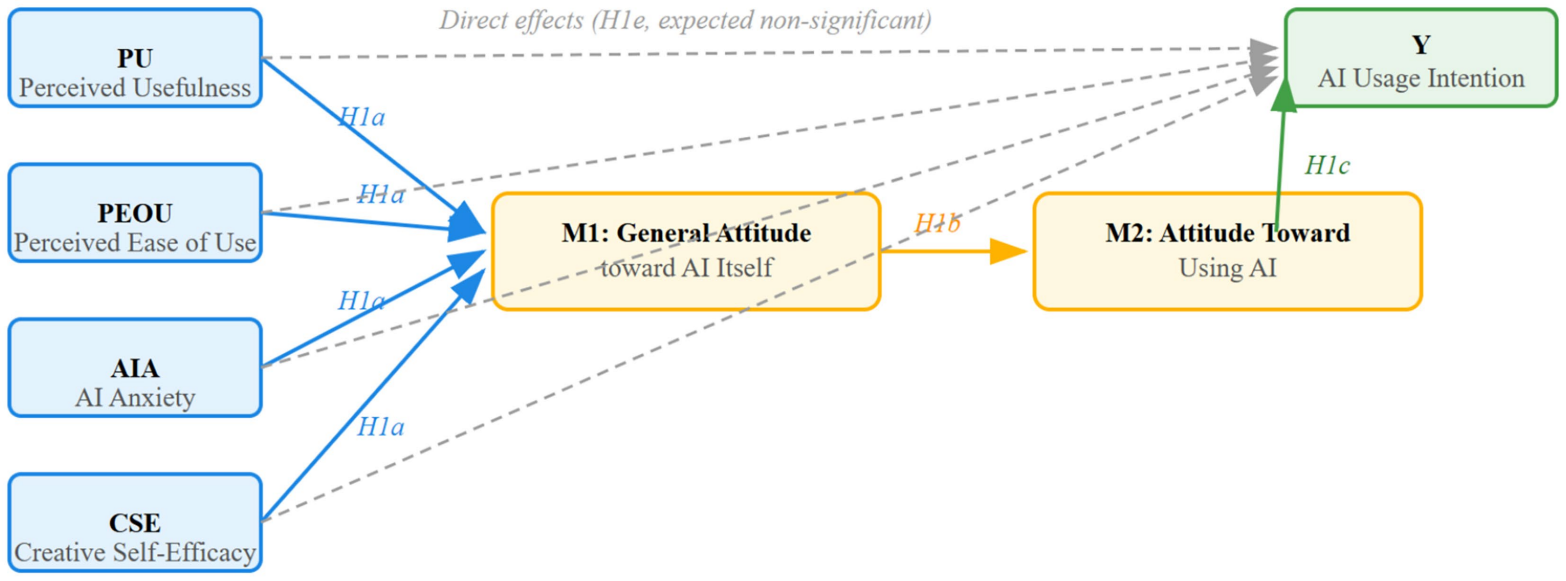
Conceptual serial mediation model. PU = perceived usefulness; PEOU = perceived ease of use; AIA = AI anxiety; CSE = creative self-efficacy; M1 = general attitude toward AI itself; M2 = attitude toward using AI; Y = AI usage intention. Solid arrows indicate the hypothesized direct and indirect paths. Specifically, paths from distal antecedents (PU, PEOU, AIA, CSE) to M1 correspond to H1a; the path from M1 to M2 corresponds to H1b; the path from M2 to Y corresponds to H1c. The serial indirect effect (X → M1 → M2 → Y) corresponds to H1d.

Based on the theoretical analysis above, we propose a serial mediation model (see Figure 1). In this model, perceived usefulness (PU), perceived ease of use (PEOU), AI anxiety (AIA), and creative self-efficacy (CSE) serve as distal antecedents. They are hypothesized to influence AI usage intention sequentially through general attitude toward AI itself (M1) and attitude toward using AI (M2). The model specifies both direct paths from each antecedent to usage intention (expected to be non-significant) and indirect paths via the two attitudinal mediators, including the serial pathway (X → M1 → M2 → Y).

Based on this conceptual model, the present study, targeting Chinese university students majoring in art and design, aims to achieve the following research objectives:

1. To test the serial mediation relationships among psychological variables – specifically, to examine whether the effects of PU, PEOU, AI anxiety, and CSE on AI usage intention are mediated by general attitude toward AI itself and attitude toward using AI, both individually and serially. This tests the core premise that these antecedents operate through a shared attitudinal chain rather than as independent predictors.
2. To reveal the heterogeneity in AI usage intention among students with different psychological characteristics and backgrounds, identify potential group types, and explore the characteristic differences in demographic and psychological variables across these groups.

To achieve these objectives, this study employs the “task allocation method” for refined measurement of AI usage intention (covering five design stages: creative ideation, material collection, visual element design, copywriting, and final modification/optimization). The study comprehensively utilizes multiple regression analysis (to test variable relationships), serial mediation analysis (PROCESS Model 6) to test the hypothesized indirect effects, and latent profile analysis (to identify heterogeneous groups) for data processing.

Accordingly, the following research hypotheses are proposed:

*H1a*: PU, PEOU, AI anxiety, and CSE significantly influence general attitude toward AI itself.

*H1b*: General attitude toward AI itself significantly influences attitude toward using AI.

*H1c*: Attitude toward using AI significantly influences AI usage intention.

*H1d*: The effects of PU, PEOU, AI anxiety, and CSE on AI usage intention are serially mediated by general attitude toward AI itself and attitude toward using AI. Specifically, the serial indirect pathway (X → M1 → M2 → Y) is significant for each antecedent, and this sequential path constitutes the primary mechanism of influence.

*H1e*: After controlling for the serial mediators, the direct effects of PU, PEOU, AI anxiety, and CSE on AI usage intention are expected to be non-significant, confirming that these variables do not function as independent predictors.

*H2*: There is significant heterogeneity in students’ AI usage intention, and two or more latent profiles with different usage patterns can be identified.

## 2 Method

### 2.1 Participants

The participants in this study were Chinese university students majoring in art and design. Data were collected through an online questionnaire distributed via professional communities and university contacts. A total of 721 questionnaires were collected. To ensure data quality, invalid samples were screened based on a trap question embedded in the questionnaire (requiring participants to select “neutral”). After excluding 91 samples that did not answer as required, 630 valid samples were obtained, yielding an effective response rate of 87.4%.

The demographic characteristics and artificial intelligence (AI) usage status of the valid sample are shown in Table 1. Regarding gender distribution, there were 165 males (26.2%) and 465 females (73.8%), with a male-to-female ratio of approximately 1:2.8, which aligns with the current situation where female students constitute a higher proportion in art and design majors. Regarding grade distribution, there were 114 freshmen (18.1%), 259 sophomores (41.1%), 238 juniors (37.8%), 13 seniors (2.1%), and 6 graduate students (1.0%). The sample was predominantly composed of sophomores and juniors, together accounting for 78.9%. The distribution of majors covered the main directions of art and design, with Visual Communication Design having the largest number (185, 29.4%), followed by Digital Media Art (133, 21.1%) and Environmental Design (131, 20.8%). Other majors such as Product Design, Arts and Crafts, Animation, Public Art, and Advertising were also involved, indicating a relatively comprehensive professional composition of the sample.

**Table 1:** Demographic characteristics and AI usage status of respondents.

Statistical dimension	Category	*N*	%
Gender	Male	165	26.2
	Female	465	73.8
Grade	Freshman	114	18.1
	Sophomore	259	41.1
	Junior	238	37.8
	Senior	13	2.1
	Graduate student	6	1
Major	Visual communication design	185	29.4
	Environmental design	131	20.8
	Product design	48	7.6
	Digital media art	133	21.1
	Arts and crafts	52	8.3
	Animation	20	3.2
	Public art	33	5.2
	Advertising	28	4.4
AI usage experience	Never used	118	18.7
	Occasionally use	296	47
	Often use	178	28.3
	Use almost daily	38	6
AI proficiency	Completely unable	76	12.1
	Beginner	337	53.5
	Intermediate	199	31.6
	Very proficient	18	2.9

Descriptive statistics for age showed that the sample age ranged from 17 to 28 years, with an average age of 20.28 years (*SD* = 1.27). The age distribution was concentrated and consistent with the grade distribution characteristics, indicating that the sample mainly consisted of lower and middle-level undergraduate students.

In terms of AI usage, 118 students (18.7%) reported “never using” AI, 296 (47.0%) “occasionally use,” 178 (28.3%) “often use,” and 38 (6.0%) “use almost daily.” Over 80 % (81.3%) of the students had experience using AI, indicating that AI tools are relatively common among art and design students. Regarding self-assessed AI proficiency, 12.1% of students reported being “completely unable to use,” 53.5% were “beginners,” 31.6% reached “intermediate proficiency,” and only 2.9% rated themselves as “very proficient.” The overall proficiency level leaned toward beginner to intermediate, suggesting that students’ mastery of AI tools is still in a developmental stage with considerable room for improvement.

In summary, the valid sample of this study covers Chinese university art and design students with diverse genders, grades, professional backgrounds, and AI usage experiences, demonstrating good representativeness and providing a reliable data foundation for subsequent analyses.

### 2.2 Measurements

This study used a self-developed questionnaire as the data collection tool. The questionnaire consisted of four parts: demographic variables, psychological mechanism variables, a scale for general attitudes toward AI itself, and the core dependent variable—AI usage intention measured by the task allocation method. All scales were adapted based on existing mature research and appropriately adjusted for the context of art and design. All psychological variables were measured using a 5-point Likert scale (1 = Strongly Disagree, 5 = Strongly Agree).

#### 2.2.1 General attitudes toward AI itself

This section was adapted from the study by Alsuwaida and Alsamiri (2025) and comprised 20 items. Some items were reverse-scored and were recoded before data analysis to ensure that higher scores represented more positive attitudes. Exploratory factor analysis results showed that the scale extracted three common factors with eigenvalues greater than 1 in this sample, with a cumulative variance contribution rate of 54.923%. Item factor loadings ranged from 0.420 to 0.849, indicating good construct validity. Inspection of the rotated component matrix revealed that Factor 1 mainly comprised items reflecting positive evaluations of AI; Factor 2 included items related to both concerns about AI replacing humans and recognition of AI’s artistic legitimacy; and Factor 3 consisted of items capturing personal threats and identity anxiety. Because the present study focuses on students’ overall attitude toward AI as a meta-cognitive disposition, and the full scale demonstrated good reliability (Cronbach’s *α* = 0.842), the total score was used in subsequent analyses.

#### 2.2.2 Psychological mechanism variables

Perceived Usefulness (PU) and Perceived Ease of Use (PEOU) scales were adapted from Davis (1989) Technology Acceptance Model (TAM), each containing three items. They measured, respectively, the extent to which students believed AI design tools helped with work efficiency, work quality, and creative enhancement, and their subjective perception of the tool’s ease of operation. The Cronbach’s *α* coefficients for the two scales were 0.881 and 0.896, respectively. Factor analysis extracted a single factor for each, with cumulative variance contribution rates of 80.839 and 82.846%, indicating good construct validity.

The AI Anxiety (AIA) scale was adapted from Zhu et al. (2025). Three items were selected for this study’s context to measure students’ unease regarding over-reliance on AI, AI replacing designers, and the rapid pace of technological development. Exploratory factor analysis extracted one common factor, with a cumulative variance contribution rate of 72.752% and factor loadings ranging from 0.833 to 0.882. Cronbach’s *α* was 0.813, indicating acceptable reliability and validity.

Creative Self-Efficacy (CSE) was adapted from Tierney and Farmer (2002), containing three items measuring students’ confidence in their ability to generate ideas and solve problems in design tasks. Factor analysis extracted one common factor, with a cumulative variance contribution rate of 81.236% and Cronbach’s *α* of 0.884, indicating good construct validity.

Attitude toward using AI (ATT) was adapted from Zhu et al. (2025), containing four items measuring the extent to which students felt positively about using AI tools in design creation. Factor analysis extracted one common factor, with a cumulative variance contribution rate of 86.823% and Cronbach’s *α* of 0.949, demonstrating excellent reliability and validity.

#### 2.2.3 AI usage intention measured by task allocation

To overcome the generality of traditional Likert scales in measuring “usage intention,” this study innovatively adopted the “task allocation method” to measure students’ degree of reliance on AI across different stages of the design process. This method is based on a classic decomposition of the design process (Ambrose and Harris, 2015), dividing a design project into the following five core stages: Creative Ideation/Brainstorming, Material Collection and Reference Analysis, Visual Element Design (e.g., graphics, layout, color schemes), Copywriting (e.g., slogans, product descriptions), and Final Modification and Optimization (e.g., detail adjustments, effect enhancement). To establish content validity, the stage definitions and wording were reviewed by three experts in art design education and two experienced design practitioners. Minor wording adjustments were made based on their feedback to ensure each stage was clearly distinguishable and relevant to students’ actual learning experiences.

For each stage, participants were asked to indicate, based on their true thoughts, the percentage of work they would prefer AI to complete (0% = completed entirely by myself, 100% = completed entirely by AI; they could enter any integer between 0 and 100). Based on this, the following two indicators were constructed: Total AI Usage Intention Index: The arithmetic mean of the AI participation percentages across the five stages, reflecting students’ overall reliance on AI; Stage-Specific AI Usage Intention: The individual score for each stage, used to analyze differences in students’ acceptance of AI at different task phases.

The reliability and validity of this measure were examined. Internal consistency was acceptable (Cronbach’s *α* = 0.835). For construct validity, an exploratory factor analysis (EFA) was conducted using SPSS with principal component extraction. The Kaiser–Meyer–Olkin (KMO) measure was 0.834, and Bartlett’s test of sphericity was significant [*χ^2^*(10) = 1168.13, *p* < 0.001], indicating that the data were suitable for factor analysis. A single factor with eigenvalue greater than 1 emerged, accounting for 60.59% of the total variance. All five items loaded strongly on this factor, with loadings ranging from 0.708 to 0.850, supporting a unidimensional structure of AI usage intention across the five design stages. For convergent validity, as shown in Table 2, the total usage intention index correlated significantly with theoretically related constructs: attitude toward using AI (*r* = 0.289, *p* < 0.01) and perceived usefulness (*r* = 0.236, *p* < 0.01).

**Table 2:** Correlation matrix among variables.

Variable	1	2	3	4	5	6	7
1. General attitude toward AI itself	--						
2. Perceived usefulness	0.572**	--					
3. Perceived ease of use	0.425**	0.628**	--				
4. AI anxiety	0.270**	0.224**	0.255**	--			
5. Creative self-efficacy	0.265**	0.361**	0.480**	0.158**	--		
6. Attitude toward using AI	0.621**	0.710**	0.548**	0.168**	0.339**	--	
7. Total usage intention	0.305**	0.236**	0.138**	0.117**	0.06	0.289**	--

**p < 0.01.

### 2.3 Data analysis strategy

To test the integrated serial mediation model (Figure 1; H1a–H1e) —which posits that the four distal antecedents do not directly influence usage intention but rather operate through a sequential attitudinal chain—we employed Hayes’ PROCESS macro (Model 6) for SPSS with 5,000 bootstrap resamples. This approach allows us to simultaneously estimate the indirect effects of each antecedent through the two attitudinal mediators in sequence, rather than treating the antecedents as independent predictors of the outcome. Separate models were run for each independent variable (PU, PEOU, AIA, CSE), with general attitude toward AI itself as the first mediator (M1), attitude toward using AI as the second mediator (M2), and AI usage intention as the outcome (Y). The model specification explicitly requires the serial pathway (X → M1 → M2 → Y) to be tested for each antecedent, with direct effects (X → Y) controlled but expected to be non-significant. All analyses controlled for demographic variables, including gender, grade, major, AI usage experience, and self-rated AI proficiency (dummy-coded where appropriate). A bootstrap 95% confidence interval (CI) that does not include zero indicates a statistically significant indirect effect.

In addition to the PROCESS analysis, we conducted hierarchical multiple regression to examine the predictive effects of the variables without specifying mediation pathways, consistent with the variable-centered approach. Latent profile analysis (LPA) was used to identify heterogeneous subgroups of students based on their AI usage intentions across five design stages. All statistical analyses were performed using SPSS 26 and Mplus 8.3.

## 3 Results

### 3.1 Reliability analysis results

Before formal analysis, internal consistency reliability tests were conducted for each scale. Results showed that all scales’ Cronbach’s *α* coefficients were above 0.80, indicating that the measurement tools had good reliability in this sample (see Table 3).

**Table 3:** Reliability coefficients of scales.

Scale	Items	Cronbach’s *α*
General attitude toward AI itself	20	0.842
Perceived usefulness (PU)	3	0.881
Perceived ease of use (PEOU)	3	0.896
AI anxiety (AA)	3	0.813
Creative self-efficacy (CSE)	3	0.884
Attitude toward using AI (ATT)	4	0.949
Usage intention (total of five stages)	5	0.835

### 3.2 Scale validity test

To examine the construct validity of each psychological scale, exploratory factor analysis (EFA) was conducted on the “General Attitude Toward AI Itself” (20 items), PU (3 items), PEOU (3 items), AIA (3 items), CSE (3 items), and ATT (4 items). Principal component analysis was used to extract factors with eigenvalues greater than 1, and varimax orthogonal rotation was applied to the multi-factor scale. The Kaiser-Meyer-Olkin (KMO) measure of sampling adequacy and Bartlett’s test of sphericity were used to determine the suitability of the data for factor analysis. Results are summarized in Table 4.

**Table 4:** Summary of exploratory factor analysis results for each scale.

Scale	Items	KMO	Bartlett’s test (*χ*^2^)	df	Extracted factors	Cumulative variance (%)	Factor loading range
General attitude toward AI itself	20	0.863	5331.296***	190	3	54.923	0.420–0.849
Perceived usefulness	3	0.735	1030.565***	3	1	80.839	0.877–0.916
Perceived ease of use	3	0.748	1140.190***	3	1	82.846	0.903–0.922
AI anxiety	3	0.704	648.254***	3	1	72.752	0.833–0.882
Creative self-efficacy	3	0.737	1054.943***	3	1	81.236	0.878–0.916
Attitude toward using AI	4	0.872	2507.836***	6	1	86.823	0.921–0.941

***p < 0.001.

The analysis results showed that the KMO values for each scale ranged from 0.704 to 0.872, and Bartlett’s tests were all significant (*p* < 0.001), indicating that the data were suitable for factor analysis. Among them, the five scales (PU, PEOU, AIA, CSE, ATT) each extracted one common factor with eigenvalue greater than 1, with cumulative variance contribution rates ranging from 72.752 to 86.823%, and all item factor loadings were above 0.8. These indicate that these scales possess ideal unidimensional structures and good construct validity.

For the “General Attitude Toward AI Itself” scale, factor analysis extracted three common factors with eigenvalues greater than 1, with a cumulative variance contribution rate of 54.923%. After varimax rotation (converging after 7 iterations), the loadings of items on their respective factors were all above 0.4 (lowest 0.420), and the factor structure was clear, indicating that this scale has a clear multidimensional structure. Considering that this study aims to examine the impact of students’ general attitude toward AI as an overall psychological construct on usage intention, and given the good overall reliability of the scale (Cronbach’s *α* = 0.842), the total scale score was used as the measurement indicator in subsequent analyses.

In summary, all psychological scales used in this study demonstrated good construct validity. The measurement tools are reliable and valid, suitable for subsequent statistical analysis.

### 3.3 Descriptive statistics

The means, standard deviations, skewness, and kurtosis for each core variable are shown in Table 5. The absolute values of skewness for all variables were less than 2, and the absolute values of kurtosis were less than 7, meeting the assumptions of normal distribution and suitable for parametric tests.

**Table 5:** Descriptive statistics of variables.

Variable	*M*	*SD*	Skewness	Kurtosis
General attitude toward AI itself	3.14	0.38	0.83	5.84
Perceived usefulness	3.59	0.69	0.22	0.25
Perceived ease of use	3.41	0.68	0.46	0.36
AI anxiety	3.25	0.74	0.1	0.92
Creative self-efficacy	3.27	0.64	0.68	1.36
Attitude toward using AI	3.42	0.71	0.1	1.19
Total usage intention	50.99	17.86	0.01	0.2

Total Usage Intention is the mean AI participation percentage across the five design stages (range 0–100).

Descriptive statistics for AI usage intention across the five design stages are shown in Table 6. The intention was highest for the Copywriting stage (*M* = 58.19, *SD* = 22.39), followed by the Material Collection stage (*M* = 54.64, *SD* = 23.35), while the intention was lowest for the Final Modification and Optimization stage (*M* = 46.12, *SD* = 24.73). This distribution suggests that students are more inclined to introduce AI in executive, auxiliary tasks like copywriting and material collection, while still preferring to maintain human dominance in core creative stages (such as creative ideation and visual element design).

**Table 6:** Descriptive statistics of AI usage intention by design stage.

Stage	*M*	*SD*
Creative ideation	48.21	21.7
Material collection	54.64	23.35
Visual element design	47.78	22.74
Copywriting	58.19	22.39
Final modification and optimization	46.12	24.73

### 3.4 Correlation analysis

Pearson correlation analysis was conducted on all variables, and the results are shown in Table 2. PU, PEOU, general attitude toward AI itself, and attitude toward using AI were all significantly positively correlated with total usage intention (*p* < 0.01). AI anxiety was also significantly positively correlated with attitude toward using AI and total usage intention (*p* < 0.01), but the direction was not entirely consistent with theoretical expectations, potentially reflecting students’ ambivalent mindset of both anxiety about and reliance on AI. Notably, the correlation coefficient between attitude toward using AI and total usage intention was 0.289 (*p* < 0.01), and between PU and total usage intention was 0.236 (*p* < 0.01), providing a basis for subsequent mediation analysis.

### 3.5 Hierarchical regression analysis

Before presenting the regression results, we first diagnosed potential multicollinearity. To address the potential concern—particularly the high correlation (*r* = 0.710) between perceived usefulness (PU) and attitude toward using AI (ATT)—we computed the Variance Inflation Factor (VIF) for all predictors in the final regression model (Model 2). The results showed that all VIF values were well below the conventional threshold of 5, ranging from 1.163 to 2.599, with the highest VIF being 2.599 for PU. Therefore, multicollinearity does not threaten the stability of our regression estimates.

To explore the predictive effects of various factors on total usage intention, hierarchical multiple linear regression analysis was used. Demographic variables (gender, grade, major, usage experience, proficiency) were entered in Step 1 as control variables. Core psychological variables (general attitude toward AI itself, PU, PEOU, AIA, CSE, ATT) were entered in Step 2. Results are shown in Table 7.

**Table 7:** Hierarchical regression analysis predicting total usage intention.

Variable	Model 1 (*β*)	Model 2 (*β*)
Control variables		
Gender	−0.127**	−0.061
Usage experience	0.119*	0.09
Proficiency	0.018	0.026
Sophomore (dummy)	0.061	0.034
Junior (dummy)	0.122*	0.094
Senior (dummy)	0.003	0.001
Graduate student (dummy)	0.04	−0.007
Environmental design (dummy)	0.065	0.121**
Product design (dummy)	0.016	0.008
Digital media art (dummy)	−0.059	−0.076
Arts and crafts (dummy)	0.065	0.113**
Animation (dummy)	−0.026	−0.033
Public art (dummy)	0.017	0.035
Advertising (dummy)	−0.011	0.011
Psychological variables		
General attitude toward AI itself	--	0.173**
Perceived usefulness	--	0.055
Perceived ease of use	--	−0.091
AI anxiety	--	0.069
Creative self-efficacy	--	−0.065
Attitude toward using AI	--	0.211***
*R* ^2^	0.056	0.164
Δ*R*^2^	--	0.108***
*F*	2.622**	5.990***

β represents standardized regression coefficients; *p < 0.05, **p < 0.01, ***p < 0.001.

After controlling for demographic variables, the variables in Step 2 significantly increased the explained variance in total usage intention [Δ*R*^2^ = 0.108, *F*(6, 609) = 13.125, *p* < 0.001]. In the final model, general attitude toward AI itself (*β* = 0.173, *t* = 3.369, *p* = 0.001) and attitude toward using AI (*β* = 0.211, *t* = 3.592, *p* < 0.001) significantly and positively predicted usage intention. Additionally, Environmental Design (*β* = 0.121, *t* = 2.651, *p* = 0.008) and Arts and Crafts (*β* = 0.113, *t* = 2.660, *p* = 0.008) also significantly predicted usage intention. PU, PEOU, AIA, and CSE were not significant in Model 2 (*p* > 0.05).

### 3.6 Serial mediation analysis

To test the integrated serial mediation model (Figure 1; H1a–H1e)—which posits that the four distal antecedents (PU, PEOU, AIA, and CSE) do not directly influence AI usage intention but rather operate through a sequential attitudinal chain (X → M1 → M2 → Y)—we conducted PROCESS Model 6 analyses for PU, PEOU, AIA, and CSE separately. This model specification explicitly tests the indirect effects of each antecedent through the two mediators in sequence, rather than treating them as independent predictors of the outcome. Table 8 summarizes the total, direct, and indirect effects (unstandardized coefficients with bootstrap 95% CIs).

**Table 8:** Serial mediation results for PU, PEOU, AIA, and CSE.

*X*	Total effect (c)	Direct effect (c’)	Total indirect	Ind1: X → M1 → Y	Ind2: X → M2 → Y	Ind3: X → M1 → M2 → Y
PU	6.09***	0.06	6.03 [3.67, 8.43]	2.78 [1.24, 4.39]	2.46 [0.83, 4.30]	0.78 [0.25, 1.38]
PEOU	3.16**	−2.06	5.22 [3.39, 7.40]	2.20 [1.01, 3.57]	1.96 [0.87, 3.44]	1.05 [0.51, 1.74]
AIA	3.04**	1.18	1.86 [0.81, 3.04]	1.17 [0.42, 2.10]	0.01 [−0.46, 0.48]	0.68 [0.26, 1.23]
CSE	1.06	−2.1	3.16 [1.81, 4.68]	1.37 [0.46, 2.56]	1.04 [0.39, 1.87]	0.75 [0.30, 1.28]

Values are unstandardized coefficients. Bootstrap 95% confidence intervals are in brackets. **p < 0.01, ***p < 0.001. Ind1 = X → general attitude (M1) → usage intention; Ind2 = X → usage attitude (M2) → usage intention; Ind3 = X → M1 → M2 → usage intention (serial indirect effect).

As shown in Table 8, for PU, PEOU, and CSE, all three specific indirect effects were significant (95% CIs excluding zero), while the direct effects were non-significant. For AIA, the indirect effect via M2 only (Ind2) was non-significant (95% CI [−0.46, 0.48]), but the indirect effects via M1 only (Ind1) and via the serial pathway (Ind3) were significant. Critically, supporting the core premise of our integrated model, the serial indirect effect (X → M1 → M2 → Y) was significant for all four independent variables (see Ind3 in Table 8). This finding supports H1d and demonstrates that the influence of PU, PEOU, AIA, and CSE on usage intention is channeled through a common sequential mechanism: first shaping general attitude toward AI itself (M1), then attitude toward using AI (M2), and finally affecting usage intention (Y).

Notably, for PU, PEOU, and CSE, the direct effects (c′) were non-significant, while the serial indirect effects were significant—a pattern of full serial mediation. For AIA, the non-significant Ind2 combined with significant Ind1 and Ind3 indicates that its effect on usage intention operates primarily through general attitude toward AI itself and the serial pathway, rather than through attitude toward using AI alone. The total effect of CSE on usage intention was not significant, yet its total indirect effect was significant—an indirect-only mediation pattern (i.e., the direct negative effect was canceled by positive indirect effects). Collectively, these results confirm that the four antecedents do not function as independent predictors; instead, their effects are fully transmitted through the sequential attitudinal chain, with general attitude toward AI itself serving as a critical gateway.

### 3.7 Latent profile analysis

The preceding variable-centered analysis revealed the mechanisms influencing usage intention. However, art and design students’ intentions to use AI design tools may exhibit qualitative heterogeneity—different student groups might show completely different patterns in their stage preferences and reliance on AI. To identify these potential subgroups, Latent Profile Analysis (LPA) was conducted using the AI usage intention scores for the five design stages (Creative Ideation, Material Collection, Visual Element Design, Copywriting, Final Modification and Optimization) as indicator variables, exploring different usage patterns within the sample and examining differences in demographic and psychological variables across profiles.

Consistent with recent person-centered research in adjacent fields (Chen and Zare, 2025), we anticipated heterogeneous subgroups of AI usage intention. Based on prior technology acceptance literature, we expected to identify profiles differing in their overall level of AI reliance across design stages, ranging from low to high, with a moderate group representing the largest segment. The three-profile solution empirically confirmed this expectation.

Latent profile analysis was estimated using Mplus 8.3 with robust maximum likelihood estimation. Models with 1 to 4 classes were fitted sequentially. Model fit indices included: Akaike Information Criterion (AIC), Bayesian Information Criterion (BIC), sample-size adjusted BIC (aBIC), Entropy, Lo–Mendell–Rubin Likelihood Ratio Test (LMR-LRT), and Bootstrap Likelihood Ratio Test (BLRT). Fit results are summarized in Table 9.

**Table 9:** Fit indices for latent profile analysis models.

Classes	AIC	BIC	aBIC	Entropy	LMR-LRT *p*	BLRT *p*	Class Counts and Proportions
1	28697.35	28741.81	28710.06	–	–	–	630(1.00)
2	28006.01	28077.14	28026.34	0.73	0.001	<0.001	253(0.40), 377(0.60)
3	27671.66	27769.47	27699.62	0.82	<0.001	<0.001	387(0.61), 134(0.21), 109(0.17)
4	27592.53	27717.01	27628.11	0.82	0.18	<0.001	136(0.22), 326(0.52), 58(0.09), 110(0.17)

As seen in Table 9, AIC, BIC, and aBIC decreased continuously as the number of classes increased, but the rate of decrease gradually slowed. The LMR-LRT was significant for the 2-class model (*p* = 0.001) and the 3-class model (*p* < 0.001), but non-significant for the 4-class model (*p* = 0.18), indicating that moving from 3 to 4 classes did not yield a statistically significant improvement in fit. Additionally, the 3-class model had a good Entropy value of 0.82, indicating good classification quality, and the smallest class proportion was 17% (109 students), meeting the requirement for class practicality.

Although the BLRT was significant for the 4-class model (*p* < 0.001), the LMR-LRT was not (*p* = 0.18), a pattern that can occur when an additional class captures only a minor distinction or when the LMR-LRT is underpowered. To make a well-informed decision, we considered multiple criteria beyond *p*-values. First, parsimony and interpretability: the 4-class model yielded a very small class comprising only 9% (*n* = 58) of the sample, which may be unstable, difficult to replicate, and less generalizable. Second, theoretical relevance: the added fourth class did not represent a theoretically novel pattern but rather split one of the existing classes (e.g., further dividing the Moderate Use group), without providing additional practical insights for art design education. Third, classification quality: although the Entropy values were identical (0.82) for the 3-class and 4-class models, the 3-class solution demonstrated clearer separation of theoretically meaningful profiles (Moderate Use Group, Low Use Group, High Use Group). Considering the preponderance of evidence—including statistical indices, parsimony, classification clarity, and theoretical interpretability—the 3-class model was selected as the final solution.

**Figure 2:**
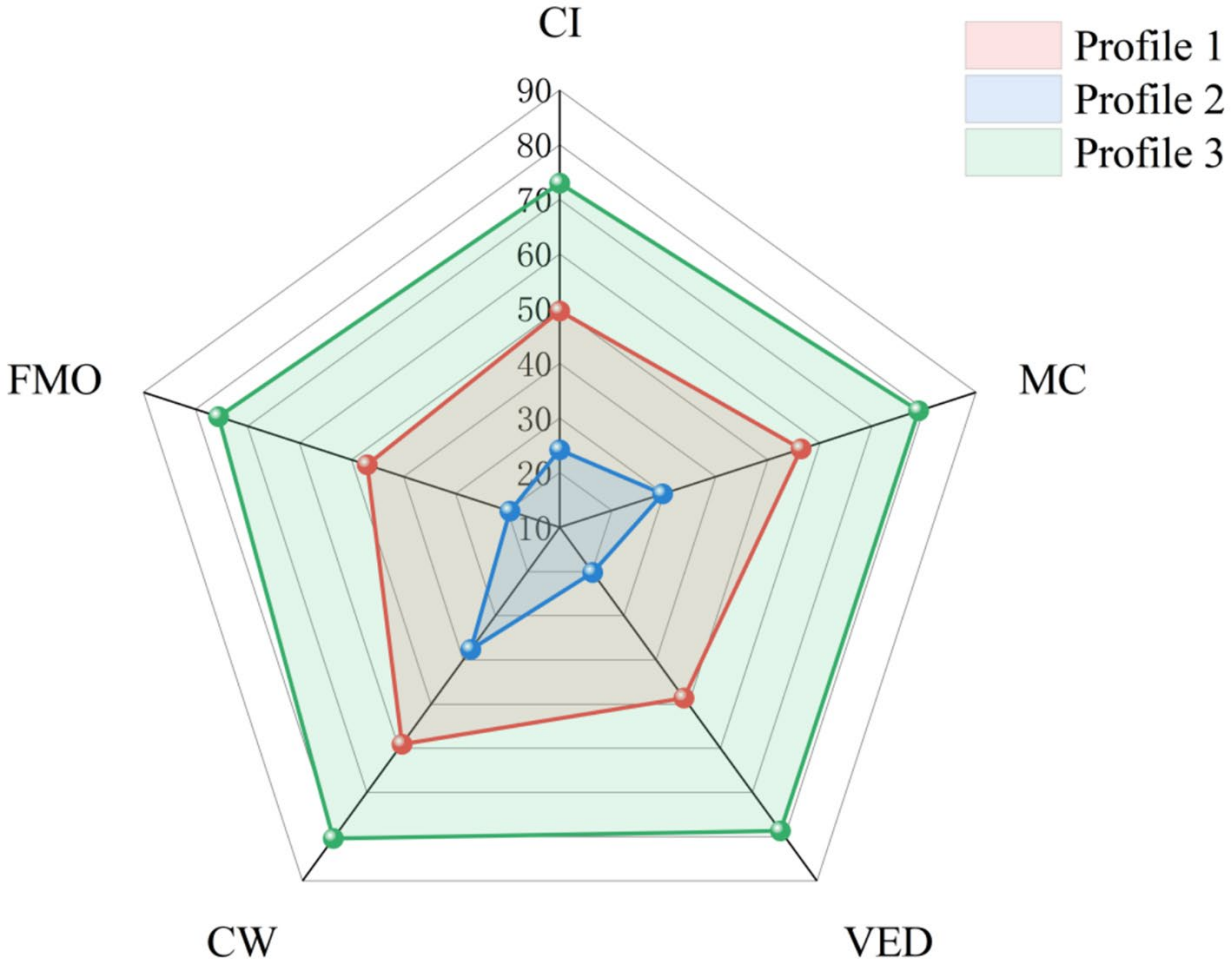
Mean scores of AI usage intention across design stages by latent profile note: CI = creative ideation; MC = material collection & reference analysis; VED = visual element design; CW = copywriting; FMO = final modification and optimization.

The conditional means of the three latent profiles on the five design stages are presented in Table 10, and the corresponding radar chart is shown in Figure 2 (based on model-estimated posterior probabilities). Based on the score patterns of AI usage intention across stages and the sample distribution, the three profiles were characterized and named as follows.

**Table 10:** AI usage intention means and sample distribution for each latent profile.

Profile	Creative Ideation	Material Collection & Reference Analysis	Visual Element Design	Copywriting	Final Modification & Optimization
Profile 1	49.55	56.4	48.62	59.05	47.02
Profile 2	24.18	29.76	20.22	37.64	19.57
Profile 3	72.97	78.99	78.66	80.39	75.55

#### 3.7.1 Profile 1: moderate use group

This profile included 387 students, accounting for 61.4% of the total sample, making it the largest group. Their AI usage intentions were at moderate levels across all design stages (means between 47 and 59). Relatively, intentions were slightly higher for Copywriting and Material Collection, and slightly lower for Visual Element Design and Final Modification and Optimization. This group tends to use AI moderately, with higher acceptance for executive and auxiliary tasks.

#### 3.7.2 Profile 2: low use group

This profile included 134 students, accounting for 21.3% of the total sample. Its defining characteristic is low AI usage intentions across all five design stages (means between 20 and 38). Among these, intention for Copywriting was relatively highest, while intentions for Visual Element Design and Final Modification & Optimization were extremely low. This group shows limited willingness to use AI, especially in core creative stages.

#### 3.7.3 Profile 3: high use group

This profile included 109 students, accounting for 17.3% of the total sample. Their AI usage intentions were at a high level across all five stages (means between 73 and 80). Intentions were particularly prominent for Copywriting, Material Collection, and Visual Element Design. Even for Creative Ideation, a stage typically considered to be primarily human-led, their intention was significantly higher than the other two profiles. This group demonstrates a strong willingness to integrate AI into all design stages.

### 3.8 Profile validity test

To verify the external validity of the three latent profiles, their differences in demographic variables and core psychological variables were further analyzed.

#### 3.8.1 Grade differences

The Kruskal-Wallis test was used to examine differences in grade distribution among the three profiles. Results showed a highly significant difference in grade distribution across profiles (*H* = 19.628, *p* < 0.001). Pairwise comparisons (see Table 11) revealed that the mean rank for grade in Profile 3 (High Use Group) (372.45) was significantly higher than that in Profile 1 (Moderate Use Group) (295.54) and Profile 2 (Low Use Group) (306.37). The difference between Profile 1 and Profile 2 was not significant (*p >* 0.05). This indicates that higher-grade students are more likely to belong to the High Use profile, while the distribution of lower-grade students is relatively balanced between the Moderate Use and Low Use profiles.

**Table 11:** Pairwise comparisons of grades across profiles.

Comparison group	Test statistic	Std. error	Std. test statistic	*p*	*Adj.p*
Profile 1 vs. Profile 2	10.83	17.023	0.636	0.525	>0.05
Profile 1 vs. Profile 3	−76.908	21.906	−3.511	<0.001	0.001
Profile 2 vs. Profile 3	−66.079	18.416	−3.588	<0.001	0.001

#### 3.8.2 Major differences

Chi-square test results showed no significant difference in major distribution among the three profiles (*χ*^2^ = 10.424, *df* = 14, *p* = 0.731). The cross-tabulation (see Table 12) shows that the distribution proportions of students from various majors across the three profiles were relatively similar, indicating that major background is not a significant factor distinguishing the three profiles. This result is also within expectations, as intention to use AI design tools is likely more influenced by individual psychological factors than by specific major orientation.

**Table 12:** Cross-tabulation of major distribution across profiles.

Major	Profile 1: moderate use group (*n* = 387)	Profile 2: low use group (*n* = 134)	Profile 3: high use group (*n* = 109)
1 (Visual Communication Design)	119 (30.7%)	36 (26.9%)	30 (27.5%)
2 (Environmental Design)	79 (20.4%)	28 (20.9%)	24 (22.0%)
3 (Product Design)	33 (8.5%)	8 (6.0%)	7 (6.4%)
4 (Digital Media Art)	81 (20.9%)	34 (25.4%)	18 (16.5%)
5 (Arts and Crafts)	28 (7.2%)	10 (7.5%)	14 (12.8%)
6 (Animation)	11 (2.8%)	4 (3.0%)	5 (4.6%)
7 (Public Art)	18 (4.7%)	7 (5.2%)	8 (7.3%)
8 (Advertising)	18 (4.7%)	7 (5.2%)	3 (2.8%)

#### 3.8.3 Differences in psychological variables

One-way ANOVA was used to test differences in core psychological variables among the three profiles, with LSD post-hoc tests. Tests of homogeneity of variances indicated that variances were unequal for PU (Levene = 4.246, *p* = 0.015), but ANOVA is generally robust with larger sample sizes. PEOU (*p* = 0.192), AIA (*p* = 0.366), and ATT (*p* = 0.068) met the homogeneity of variances assumption.

ANOVA results showed significant differences among the three profiles on all four psychological variables: Perceived Usefulness, *F*(2,627) = 13.258, *p* < 0.001; Perceived Ease of Use, *F*(2,627) = 4.934, *p* = 0.007; AI Anxiety, *F*(2,627) = 3.531, *p* = 0.030; and Attitude Toward Using AI, *F*(2,627) = 20.512, *p* < 0.001 (see Table 13).

**Table 13:** Means and standard deviations of core psychological variables across profiles.

Variable	Profile 1: moderate use group (*n* = 387)	Profile 2: low use group (*n* = 134)	Profile 3: high use group (*n* = 109)	*F*(2,627)	*p*	*Post-hoc* (LSD)
Perceived usefulness	3.60 (0.66)	3.37 (0.63)	3.82 (0.81)	13.258	<0.001	3 > 1 > 2
Perceived ease of use	3.39 (0.65)	3.32 (0.69)	3.59 (0.74)	4.934	0.007	3 > 1, 3 > 2
AI anxiety	3.26 (0.70)	3.13 (0.82)	3.38 (0.73)	3.531	0.03	3 > 2
Attitude toward using AI	3.43 (0.64)	3.14 (0.76)	3.71 (0.74)	20.512	<0.001	3 > 1 > 2

*Post-hoc* comparisons based on LSD method, significance level 0.05. 1 = Moderate Use Group, 2 = Low Use Group, 3 = High Use Group. “>“indicates significantly higher score.

Post-hoc comparisons (LSD) further revealed that for Perceived Usefulness, all three profiles differed significantly from each other (*p* < 0.01). Profile 3 (High Use Group) had the highest PU (*M* = 3.82), significantly higher than Profile 1 (*M* = 3.60) and Profile 2 (*M* = 3.37), and Profile 1 was significantly higher than Profile 2. For Perceived Ease of Use, Profile 3’s PEOU (*M* = 3.59) was significantly higher than Profile 1’s (*M* = 3.39, *p* = 0.009) and Profile 2’s (*M* = 3.32, *p* = 0.003), while the difference between Profile 1 and Profile 2 was not significant (*p* = 0.295). For AI Anxiety, Profile 3’s AIA (*M* = 3.38) was significantly higher than Profile 2’s (*M* = 3.13, *p* = 0.009), but the differences between Profile 1 and Profile 2 (*p* = 0.074) and between Profile 1 and Profile 3 (*p* = 0.139) were not significant. For Attitude Toward Using AI, all three profiles differed significantly from each other (*p* < 0.001). Profile 3 had the most positive attitude (*M* = 3.71), significantly higher than Profile 1 (*M* = 3.43) and Profile 2 (*M* = 3.14), and Profile 1 was significantly higher than Profile 2.

## 4 Discussion

This study investigated Chinese university students majoring in art and design, adopting a combined variable-centered and person-centered approach to systematically examine the psychological mechanisms and heterogeneity of students’ AI usage intentions. At the variable-centered level, hierarchical regression analysis tested the predictive effects of psychological variables—including perceived usefulness, perceived ease of use, AI anxiety, creative self-efficacy, general attitudes toward AI itself, and attitudes toward using AI—on AI usage intention. At the person-centered level, latent profile analysis based on AI usage intentions across five design stages identified three heterogeneous group types, and characteristic differences in demographic and psychological variables across profiles were examined. The following section provides an in-depth discussion of the research findings and elaborates on their theoretical contributions and practical implications.

### 4.1 Predictive effects of psychological variables on AI usage intention

This study found that, after controlling for demographic variables, general attitude toward AI itself and attitude toward using AI were significant positive predictors of art and design students’ AI usage intention, while the predictive effects of perceived usefulness, perceived ease of use, AI anxiety, and creative self-efficacy were not significant. This result aligns with some existing studies and also reveals the specificities of the art design education context.

The significant positive prediction of usage intention by general attitude toward AI itself echoes the findings of Xue (2026), namely, that the more positive students’ overall attitude toward AI, the higher their visual literacy regarding AI-generated images and their usage intention. As Alsuwaida and Alsamiri (2025) pointed out, general attitude toward AI itself encompasses individuals’ identification with the value of AI-generated art, their cognition of AI ethics and copyright issues, and their understanding of the relationship between AI and human creativity—a more stable, abstract meta-cognitive disposition. In the context of art design education, students’ acceptance of AI depends not only on the functional attributes at the tool level but is more deeply influenced by their judgment of AI’s artistic value. The experimental study by Hong and Curran (2019) similarly showed that participants holding the belief that “AI cannot create art” rated AI artworks significantly lower. Therefore, cultivating students’ positive attitudes toward AI itself, enabling them to recognize its reasonable positioning and value in creative work, may be an effective way to enhance their usage intention.

Attitude toward using AI significantly and positively predicted usage intention, consistent with findings by Zhu et al. (2025), where attitude was identified as a key mediating variable influencing behavioral intention. According to the basic logic of the Theory of Reasoned Action and the Technology Acceptance Model, the more positive an individual’s attitude toward a specific behavior, the stronger their behavioral intention (Davis et al., 1989). In this study, attitude toward using AI measured the extent to which students felt positively about using AI tools in design creation. This variable had the highest standardized coefficient in the regression model (*β* = 0.211), indicating its core role in explaining students’ AI usage intention. It is worth noting that this variable itself may be influenced by cognitive variables such as perceived usefulness and perceived ease of use, as well as affective variables like AI anxiety. Future research could further explore its antecedents and mediating mechanisms.

Drawing on self-determination theory (Ryan and Deci, 2000), the non-significant effects of perceived usefulness and perceived ease of use in the regression model represent a theoretically meaningful departure from classic TAM predictions. According to this theory, creative work satisfies intrinsic needs for autonomy and competence. When AI tools are perceived as encroaching on these needs—even if highly useful—they may be met with resistance rather than acceptance. Rather than merely reflecting statistical artifacts or insufficient power, these null findings point to domain-specific boundary conditions rooted in the unique epistemic culture of art and design education. Unlike utilitarian task contexts where PU and PEOU directly drive usage intention, creative domains are characterized by a fundamental tension between “tool empowerment” and “identity threat” (Hwang and Wu, 2025). Students may perceive highly useful and easy-to-use AI tools not as unqualified assets but as potential threats to their creative ownership and professional identity. Consistent with this interpretation, Fan and Jiang’s (2024) study on designers’ continuance intention to use AI drawing tools also found that perceived ease of use had no significant effect, and Zhou et al. (2024) similarly reported that perceived usefulness had no significant effect on behavioral intention in an AI-enhanced design acceptance model. Based on this reasoning, a testable hypothesis for future research is that the positive effect of PU on AI usage intention is moderated by perceived creative ownership, such that the effect is weaker when students believe AI diminishes their personal authorship over the final design outcome. Additionally, PEOU may negatively predict AI usage intention among students with high creative self-efficacy, as these students might interpret operational difficulty as a signal of professional legitimacy and creative depth.

AI anxiety showed a significant positive correlation with total usage intention in the correlation analysis, but was not significant in the regression model. This pattern—a significant zero-order correlation but a non-significant direct effect after controlling for attitudes—suggests that anxiety’s influence is fully mediated by cognitive appraisals (e.g., attitude toward AI itself). More importantly, the positive correlation challenges the intuitive assumption that anxiety uniformly inhibits technology adoption. Drawing on the Yerkes-Dodson law (Yerkes and Dodson, 1908), which posits an inverted U-shaped relationship between arousal and performance, we interpret this as evidence that moderate AI anxiety may enhance vigilance and motivated engagement, whereas low anxiety reflects indifference and high anxiety leads to avoidance. In the context of art and design education, we interpret this as evidence that moderate AI anxiety may signal students’ awareness of industry shifts and their motivation to stay competitive, thereby stimulating rather than suppressing usage intention. This aligns with Hwang and Wu’s (2025) observation that anxiety was significantly positively correlated with creative cognition, and with Ağca and Korkmaz’s (2025) finding that AI anxiety is a complex construct with different facets that can have opposing effects. A testable hypothesis arising from this interpretation is that AI anxiety follows an inverted U-shaped relationship with AI usage intention among art and design students, such that moderate levels promote intention by enhancing perceived relevance and urgency, while very low or very high levels inhibit it through complacency or avoidance, respectively. This hypothesis can be tested using polynomial regression or spline models.

Creative self-efficacy was significantly positively correlated with attitude toward using AI in the correlation analysis, but its correlation with total usage intention was not significant, and it was not significant in the regression model. This pattern—a strong association with the mediator but no total effect on the outcome—indicates what we term a competence–reliance paradox. According to social cognitive theory (Bandura, 1977), self-efficacy comprises both efficacy expectations (belief in one’s capability) and outcome expectations (belief that a behavior will produce a desired outcome). In the context of AI adoption, high creative self-efficacy may simultaneously lower the instrumental need for external assistance (a negative direct path) through strong efficacy expectations, while fostering positive evaluations of novel tools (a positive indirect path) through openness to innovation and reduced threat perception. Students with higher creative self-efficacy may possess stronger confidence in their own ideation and execution abilities, which could reduce their instrumental need for AI assistance—even as they hold favorable attitudes toward the technology. In other words, CSE may simultaneously promote positive evaluations of AI (through openness to innovation and reduced threat perception) and suppress direct usage intention (through preference for self-reliance). This interpretation is consistent with the indirect-only mediation observed in our PROCESS analyses. A directly testable hypothesis emerging from this paradox is that creative self-efficacy has a negative direct effect on AI usage intention but a positive indirect effect via attitude toward AI, resulting in a suppressed total effect. This pattern can be examined using two-stage least squares or moderated mediation models that distinguish between general CSE and AI-specific creative self-efficacy (Xue, 2026).

The non-significant direct effects of PU, PEOU, and CSE in the hierarchical regression can be explained by their full mediation through attitudinal variables, as revealed by the PROCESS analyses. Specifically, these cognitive and trait variables do not directly determine usage intention; rather, they shape students’ general attitude toward AI itself, which in turn influences their attitude toward using AI, ultimately affecting behavioral intention. This finding highlights the central role of meta-cognitive attitudes as a psychological gateway in art and design students’ AI acceptance process. For AI anxiety, the positive total effect and the significant indirect effect via general attitude suggest that moderate anxiety may paradoxically promote usage intention by enhancing students’ overall valuation of AI’s role in creative work, consistent with Hwang and Wu (2025). The indirect-only mediation for CSE—where total effect was non-significant but indirect effects were positive—indicates that high creative self-efficacy students may have a lower direct inclination to use AI (possibly due to confidence in their own abilities), yet they still develop positive attitudes toward AI, which indirectly increase their usage intention. Thus, across all three non-significant antecedents, explicit theoretical mechanisms—autonomy threat (self-determination theory), inverted-U arousal (Yerkes-Dodson law), and efficacy-outcome distinction (social cognitive theory)—provide solid grounding for the observed patterns.

Taken together, the pattern of results—non-significant direct effects alongside consistently significant serial indirect effects—validates our integrated theoretical model (Figure 1). It demonstrates that perceived usefulness, perceived ease of use, AI anxiety, and creative self-efficacy do not function as independent predictors of AI usage intention. Rather, they are distal antecedents whose influence is sequentially channeled through meta-cognitive (general attitude toward AI itself) and task-specific (attitude toward using AI) attitudes. This finding confirms the core premise of our model: that in the context of art and design education, the effects of cognitive, emotional, and trait variables on behavioral intention are transmitted via a shared attitudinal chain, rather than operating independently.

### 4.2 Characteristics of heterogeneous groups in students’ AI usage intention

Latent profile analysis identified three groups with distinct AI usage patterns: Moderate Use Group (61.4%), Low Use Group (21.3%), and High Use Group (17.3%). This result supports research hypothesis H2, verifying significant heterogeneity in art and design students’ AI usage intentions. The three groups showed qualitative differences in their AI usage intention score patterns across the five design stages, and exhibited expected systematic differences in psychological variables and grade distribution across profiles, confirming the external validity of the profiles.

The High Use group showed extremely high AI usage intentions across all five design stages, with intentions significantly higher than the other two profiles even for Creative Ideation, a stage typically considered human-led. This group had the highest proportion of higher-grade students, scored highest on perceived usefulness, perceived ease of use, and attitude toward using AI, and had moderate levels of AI anxiety. The characteristics of this group are highly similar to the “interested and engaged” group identified by Jiang et al. (2026), which was characterized by high intrinsic motivation and high work engagement, scoring significantly higher than the other group on all technology acceptance variables. Chen and Zare's (2025) study also identified a “high engagement thinkers” profile (33.0%), showing strong cognitive engagement related to AI. The existence of the High Use group indicates that some students already regard AI as an integral part of creative work, willing to extensively integrate AI into all stages of the design process, including the ideation phase. The moderate level of AI anxiety in this group, rather than the lowest, suggests that moderate anxiety may not hinder their usage intention but rather coexists with positive acceptance, reflecting the complex tension between “tool empowerment” and “identity threat” (Hwang and Wu, 2025).

The Moderate Use group was the largest, with AI usage intentions at moderate levels across all design stages, slightly higher for executive, auxiliary tasks like Copywriting and Material Collection, and slightly lower for core stages like Visual Element Design and Final Modification and Optimization. This group had middle-range grade distribution and scores on psychological variables, reflecting the rational acceptance attitude of pragmatists. The existence of this group validates the study’s theoretical expectation that most students position AI as a practical tool to enhance work efficiency, tending to use it moderately in executive tasks, while still preferring to maintain human dominance in stages involving core visual expression. This pattern echoes the findings of Bao et al. (2026), where compatibility and relative advantage were key factors influencing design students’ continuance intention to use generative AI, with students focusing more on the fit between AI tools and their existing design workflows.

The Low Use group showed low AI usage intentions across all five design stages, especially unwilling to use AI in core creative stages like ideation and visual design. This group had a higher proportion of lower-grade students, scored lowest on perceived usefulness and attitude toward using AI, and also had lower AI anxiety. This characteristic suggests that low use may not stem from strong AI anxiety, but rather from lack of usage experience, limited understanding of AI’s capabilities, or low confidence in their own creative abilities. Wang (2024) study found that education level was significantly positively correlated with attitude toward AI painting technology, with higher education level students having more positive attitudes. The Low Use group in this study, predominantly lower-grade students, aligns with the characteristics of their developmental stage. Notably, this group’s AI anxiety score was the lowest, which might seem counterintuitive. A possible explanation is that this group’s low usage intention is not driven by anxiety about AI, but rather by limited exposure and experience with AI tools. Students with lower AI usage experience and proficiency were more likely to have lower usage intentions. Without sufficient hands-on experience, these students may not have developed strong anxiety, yet they also lack the perceived utility or confidence to adopt AI extensively. This interpretation aligns with a sequential pathway whereby familiarity precedes both anxiety and usage intention, rather than anxiety directly suppressing use. This finding suggests that the formation mechanism of the Low Use type may differ from technology resistance driven by high anxiety, and future research should further examine the causal direction between AI anxiety and usage intention using longitudinal designs.

No significant differences were found in major distribution across the three groups, indicating that AI design tool usage intention is more influenced by individual psychological traits and developmental stage than by major background. This result aligns with findings by Jiang et al. (2026), where art and design practitioners could be divided into different subgroups based on intrinsic motivation and work engagement, with major background not being a significant differentiating factor. At the same time, this finding provides a basis for developing general teaching strategies in art design education, suggesting that entirely different AI integration plans based solely on major differences may not be necessary, and more attention should be paid to students’ psychological characteristics and developmental stages.

### 4.3 Practical implications for art design education

This study provides important practical implications for integrating AI tools into art design education. First, educators should focus on cultivating students’ meta-cognitive attitudes toward AI, moving beyond mere operational skills training to guide students in deeply reflecting on AI’s artistic value, ethical boundaries, and its relationship with human creativity. Through curriculum design that incorporates discussions on AI ethics and value analysis, educators can help students develop rational, open, and critical attitudes toward AI. At the same time, attention should be paid to the complexity and dynamism of students’ AI anxiety, distinguishing between different facets such as learning anxiety and job replacement anxiety, and adopting targeted strategies: on one hand, alleviate learning anxiety through clear operational guidance and ample practice; on the other hand, help students establish reasonable perceptions of the AI-human relationship through career development guidance and creative value analysis, enabling them to recognize the unique advantages of human creativity in emotional expression and cultural context understanding. To operationalize these strategies, educators can implement concrete classroom activities. For example, a 15-min “AI anxiety check-in” at the start of an AI-integrated course allows students to anonymously write down one worry about AI; the instructor then categorizes these into learning anxiety (e.g., “I do not know how to use the tool”) and job replacement anxiety (e.g., “AI will make designers unnecessary”), and responds with tailored solutions—technical tutorials for the former, and case studies of AI-augmented creative careers for the latter. Another practical tool is a “role-based anxiety response system,” where students discuss scenarios such as “AI generates a logo similar to my idea” to distinguish between legitimate concerns and overgeneralized fears.

Second, educators should identify students’ heterogeneous usage patterns and implement differentiated instruction. Based on the three latent profiles identified in this study (Moderate Use Group, Low Use Group, High Use Group), we propose the following concrete teaching strategies:

For the High Use Group, who show strong willingness to integrate AI across all design stages, provide more challenging AI-integrated tasks that encourage deep application of AI in the ideation stage, while guiding them to maintain critical reflection to prevent technological reliance from eroding originality. A specific classroom example is the “Human–AI Co-creation Reflection Task”: students first generate three design variants using AI (e.g., three poster layouts from Midjourney), then manually revise each variant and write a short reflection documenting where AI assisted or hindered their originality. This exercise fosters critical AI literacy and prevents over-reliance.

For the Moderate Use Group, the largest cohort, reinforce AI’s instrumental value in executive tasks and gradually guide them to expand into creative stages. A practical staged assignment can be designed: in Week 1–2, students use AI only for copywriting and material collection; in Week 3–4, they are encouraged to use AI for initial visual element generation; and in Week 5–6, they may optionally involve AI in creative ideation. This scaffolding approach respects their pragmatic attitude while gently expanding their comfort zone.

For the Low Use Group, who show low intention partly due to unfamiliarity rather than high anxiety, focus on building usage experience through step-by-step practical tasks to reduce technological unfamiliarity, gradually introducing AI’s design value. A concrete strategy is the “template-based AI exploration worksheet”: for a low-stakes task (e.g., generating color palettes or slogan options for a simple poster), students follow a step-by-step guided worksheet that shows exactly which prompts to enter and how to interpret outputs. After completing the task, students answer two reflection questions (“Did AI save you time?” and “Did AI offer an idea you had not thought of?”). Repeating this for three sessions significantly increases usage intention without triggering defensive reactions.

### 4.4 Limitations and future research directions

Although this study yielded several valuable findings, it has the following limitations that should be addressed in future research. First, limitations in sample representativeness and cultural specificity. The sample consisted exclusively of Chinese university students majoring in art and design. While this provides valuable insights into the Chinese higher education context, generalizing the findings to other cultural or national contexts requires substantial caution. As Bao (2025) demonstrated, cross-cultural differences in attitudes toward generative AI are significant among the US, Japan, and China, with Chinese participants showing higher acceptance and value recognition of AI-generated art, partly due to different usage experiences and cultural orientations toward technology and authorship. Moreover, China’s centralized education system and rapid AI policy promotion may shape students’ psychological mechanisms differently than in more individualistic or less technologically centralized societies. Therefore, the interpretations and implications discussed in this study should be primarily understood as applicable to the Chinese art and design education context. Future research should conduct cross-national comparative studies or cross-cultural validations to examine the universality and cultural specificity of the identified psychological mechanisms and heterogeneous profiles. Second, the cross-sectional design limits causal inference. While this approach can reveal correlations between variables, it cannot determine causal relationships. Future research could adopt longitudinal tracking designs or experimental methods to explore the causal directions and dynamic change patterns between psychological variables and usage intention. Third, limitations of measurement tools. The self-developed AI anxiety scale contained only three items and could not fully capture the multidimensional structure of AI anxiety. Future research could adopt more refined scales, such as the one developed by Wang and Wang (2022) that includes multiple dimensions like learning anxiety and job replacement anxiety, and further clarify the conceptual boundaries and theoretical relationship between attitude toward using AI and general attitude toward AI itself. Furthermore, the general attitude toward AI scale exhibited a three-factor structure in this sample, indicating that it captures multiple dimensions (positive attitudes, mixed risk-recognition, and identity threat). While the total score was used to represent an overarching attitude, future research should examine whether these sub-dimensions have distinct predictive effects on AI usage intention. Fourth, the stability of the latent profile analysis results needs verification. The three groups identified based on the sample of 630 require validation in independent samples. Future research could collect more diverse samples to test the cross-sample stability of these profiles and explore their trajectories over time. Fifth, objective behavioral indicators were not included. This study measured usage intention using the self-report task allocation method and did not examine students’ actual AI usage behavior. Future research could collect behavioral log data to explore the transformation relationship between usage intention and actual behavior, as well as differences in actual behavioral performance across different profile groups.

## 5 Conclusion

Based on a survey of 630 art and design university students, this study revealed the psychological mechanisms and group heterogeneity underlying students’ AI usage intention. Variable-centered analysis indicated that general attitude toward AI itself and attitude toward using AI were core factors significantly predicting usage intention, while traditional technology acceptance variables such as perceived usefulness did not reach significance, highlighting the importance of meta-cognitive attitudes in the art and design context. The person-centered perspective further identified three heterogeneous groups: Moderate Use Group, Low Use Group, and High Use Group, which exhibited significant differences in grade distribution and psychological characteristics. The findings provide a theoretical basis and practical implications for the differentiated integration of AI tools in Chinese art design education, while cautioning against overgeneralization to other cultural settings without further empirical validation.

## Data Availability

The datasets presented in this study can be found in online repositories. The names of the repository/repositories and accession number(s) can be found at: https://data.mendeley.com/datasets/jfcxgcmgj8/1.
